# Differentiating *Batrachochytrium dendrobatidis* and *B. salamandrivorans* in Amphibian Chytridiomycosis Using RNAScope^®^ *in situ* Hybridization

**DOI:** 10.3389/fvets.2019.00304

**Published:** 2019-09-12

**Authors:** Robert J. Ossiboff, Anastasia E. Towe, Melissa A. Brown, Ana V. Longo, Karen R. Lips, Debra L. Miller, E. Davis Carter, Matthew J. Gray, Salvatore Frasca

**Affiliations:** ^1^Department of Comparative, Diagnostic, and Population Medicine, College of Veterinary Medicine, University of Florida, Gainesville, FL, United States; ^2^Department of Biomedical and Diagnostic Sciences, College of Veterinary Medicine, University of Tennessee, Knoxville, Knoxville, TN, United States; ^3^Department of Biology, University of Florida, Gainesville, FL, United States; ^4^Department of Biology, University of Maryland College Park, College Park, MD, United States; ^5^Center for Wildlife Health, University of Tennessee Institute of Agriculture, Knoxville, TN, United States

**Keywords:** chytrid, dermal glands, frog, fungus, ISH, pathology, salamander

## Abstract

*Batrachochytrium dendrobatidis* and *B. salamandrivorans* are important amphibian pathogens responsible for morbidity and mortality in free-ranging and captive frogs, salamanders, and caecilians. While *B. dendrobatidis* has a widespread global distribution, *B. salamandrivorans* has only been detected in amphibians in Asia and Europe. Although molecular detection methods for these fungi are well-characterized, differentiation of the morphologically similar organisms in the tissues of affected amphibians is incredibly difficult. Moreover, an accurate tool to identify and differentiate *Batrachochytrium* in affected amphibian tissues is essential for a specific diagnosis of the causative agent in chytridiomycosis cases. To address this need, an automated dual-plex chromogenic RNAScope^®^ *in situ* hybridization (ISH) assay was developed and characterized for simultaneous detection and differentiation of *B. dendrobatidis* and *B. salamandrivorans*. The assay, utilizing double Z target probe pairs designed to hybridize to 28S rRNA sequences, was specific for the identification of both organisms in culture and in formalin-fixed paraffin-embedded amphibian tissues. The assay successfully identified organisms in tissue samples from five salamander and one frog species preserved in formalin for up to 364 days and was sensitive for the detection of *Batrachochytrium* in animals with qPCR loads as low as 1.1 × 10^2^ zoospores/microliter. ISH staining of *B. salamandrivorans* also highlighted the infection of dermal cutaneous glands, a feature not observed in amphibian *B. dendrobatidis* cases and which may play an important role in *B. salamandrivorans* pathogenesis in salamanders. The developed ISH assay will benefit both amphibian chytridiomycosis surveillance projects and pathogenesis studies by providing a reliable tool for *Batrachochytrium* differentiation in tissues.

## Introduction

Chytridiomycosis is a devastating fungal disease causing substantial and ongoing global amphibian biodiversity loss (1–4). Two fungal pathogens have been identified as agents of amphibian chytridiomycosis, *Batrachochytrium dendrobatidis* (*Bd*), and *B. salamandrivorans* (*Bsal*) (1, 5, 6). Both species are believed to have originated in Asia, and have been spread globally as a result of anthropogenic factors (2, 7). *Bd* was first identified in 1998 (5); it can cause clinical disease in all orders of amphibians, including frogs, salamanders, and caecilians, and can be found on all continents where amphibians occur (8).

In contrast, *Bsal* was first documented in 2013, and while it can be detected on some frog species, reports of clinical disease are restricted to salamanders (1, 9, 10). The documented distribution is limited to Asia and Europe (1, 11, 12). Given the potential for widespread *Bsal*-associated mortalities on other continents with high salamander biodiversity, like the Americas, there is a critical and urgent need for accurate, specific, and sensitive molecular and tissue-based diagnostic tests for identification of *Bsal* (2, 13). The case definition for confirmed *Bsal* chytridiomycosis includes histopathology consistent with *Bsal* infection with correlating molecular diagnostics (14). While there are some unique morphologic features particular to each *Bd* and *Bsal* in culture (15), differentiation in tissues can be difficult. As natural mixed *Bd*/*Bsal* infections are likely given the high prevalence of *Bd* in some wild American salamander populations if *Bsal* was ever to be introduced, there is a distinct need for tissue-level differentiation of *Bd* and *Bsal* (16–18).

Identification and differentiation of pathogens in tissue section are primarily achieved by either antibody- or nucleic acid-based modalities. In antibody-based assays, such as immunohistochemistry, unique protein epitopes to a target pathogen are required. Differentiation of closely related pathogens using IHC can be difficult due to conserved amino acid sequence, and polyclonal anti-*Bd* antibodies cross-react with *Bsal* (1). Even a monoclonal antibody, 5C4, specific for *Bd* and *Bsal*, binds to proteins from both organisms in western blots (19). While the western blot banding profile for each of the two organisms was unique, the antibody would be unlikely to differentiate *Bd* and *Bsal* in tissue section given its dual reactivity. In contrast, nucleic acid-based tissue-based detection modalities, namely *in situ* hybridization (ISH), utilize specific, and unique stretches of genomic or expressed RNA sequence. Recent advances in ISH, namely RNAScope^®^ technology, have improved the sensitivity and specificity of the technique as well as developing methodologies for simultaneous detection of multiple nucleic acid targets (20–22).

To simultaneously detect and differentiate *Bd* and *Bsal* organisms in formalin-fixed, paraffin-embedded skin sections from amphibians, an automated dual-plex chromogenic RNAScope^®^ ISH was developed and characterized. The specificity of the assay was tested on both pure cultures of *Bd* and *Bsal* and infected animals, the effect of formalin-fixation time and *Bd*/*Bsal* loads on ISH results was compared, and the technique was utilized to assess the distribution of *Bd* and *Bsal* in infected animals.

## Materials and Methods

### *Batrachochytrium dendrobatidis* (*Bd*) and *B. salamandrivorans* (*Bsal*) Culture

*Bd* isolate ALKL1 (isolated from an eastern newt [*Notophthalmus viridescens*] from Virginia, USA) and *Bsal* isolate AMFP (isolated from a morbid wild fire salamander [*Salamandra salamandra*] from Bunderbos, The Netherlands) were grown on 1% tryptone plates (1, 23). The cultures were individually scraped and placed in a 1.5 ml microcentrifuge tube containing approximately 0.7 ml 70% ethanol. Additional aliquots of scraped *Bd* and *Bsal* were combined and fixed in 70% ethanol together. Microcentrifuge tubes were briefly centrifuged, ethanol was removed, and the fixed fungi were suspended in Histogel (Richard-Allan Scientific, Kalamazoo, Michigan), processed routinely and embedded in paraffin.

### Experimental Infection of Eastern Newts (*Notophthalmus viridescens*) With *Bd* and *Bsal*

Adult eastern newts were collected from Maryland, screened for *Bd* and *Bsal*, treated with itraconazole, and experimentally infected with *Bd, Bsal*, or both *Bd* and *Bsal* as previously described (23). All *Batrachochytrium* infected newts died during the experiment, and uninfected control newts were euthanized using benzocaine hydrochloride. Postmortem swabs were screened for *Bd* and *Bsal* by qPCR as previously described (23). Newts were collected under MD Department of Natural Resources permit No. 56,427. Lab protocols were performed under University of Maryland IACUC protocol R-15-15.

Newts were fixed in 10% neutral buffered formalin for ~48 h before being transferred to 70% ethanol. Two newts from each experimental group (*Bd*-/*Bsal*-; *Bd*+/*Bsal*-; *Bd*-/*Bsal*+; *Bd*+/*Bsal*+) were decalcified in 0.5 M ethylenediamine tetraacetate acid (EDTA), ph 8.0 for ~24 h. Serial transverse sections of the body and sagittal sections of all limbs and the tail were taken and submitted for routine processing and paraffin embedding.

### Experimental Infection of Large-Blotched Ensatinas (*Ensatina eschscholtzii klauberi*), Red Salamanders (*Pseudotriton ruber*), Eastern Newts (*Notophthalmus viridescens*), Red-Legged False Brook Salamanders (*Aquiloeurycea cephalica*), and Yellow-Eyed Ensatinas (*Ensatina eschscholtzii xanthoptica*) With *Bsal*

Large-blotched ensatinas (*E. e. klauberi*) and red salamanders (*Pseudotriton ruber*) were raised in captivity by Indoor Ecosystems LLC (Whitehouse, OH, USA). Eastern newts (*N. viridescens*), yellow-eyed ensatinas (*Ensatina eschscholtzii xanthoptica*), and red-legged false brook salamanders (*Aquiloeurycea cephalica*) and were collected from the wild (Tennessee, USA, California, USA, and Mexico, respectively). *Notophthalmus viridescens* were collected under Tennessee Wildlife Resources Agency Scientific Collection Permit #1990. *Ensatina eschscholtzii xanthoptica* were collected under California Department of Fish and Wildlife Scientific Collection Permit #SC-11505. *Aquiloeurycea cephalica* were collected under permit from the Agriculture, Stockbreeding, Rural Development, Fishing and Food Ministry of Mexico and import permit #MA87825B-1 from the United States Fish and Wildlife Service. The *A. cephalica* were determined to be naturally infected with *Bd* by qPCR using previously described methods, which provided an opportunity for a natural co-exposure experiment (24). All other animals were verified as *Bd* qPCR negative.

*Bsal* isolate AMFP was grown on tryptone gelatin hydrolysate plates and flooded to create serial dilutions from 5 × 10^3^ to 5 × 10^6^ *Bsal* zoospores/mL. Individuals from each species were exposed to *Bsal* in 100-mL containers for 24 h that contained 1 mL of inoculum and 9 mL of autoclaved water. After the *Bsal* challenge, salamanders were housed in husbandry containers with moist paper towel and a cover object. Salamanders were humanely euthanized using benzocaine hydrochloride upon loss of righting ability. All husbandry and euthanasia procedures followed the Amphibian Husbandry Resource Guide of the Association of Zoos and Aquariums and the Guide for Euthanasia by the American Veterinary Medical Association. All animal procedures were approved by University of Tennessee Institutional Animal Care and Use Committee protocol #2395.

At necropsy, all animals were swabbed to test for the presence of *Bsal* DNA (and *Bd* for *A. cephalica* co-exposure experiments). Genomic DNA was extracted (Qiagen DNeasy Blood and Tissue Kit, Hilden, Germany) and quantitative PCR performed using an Applied Biosystems Quantstudio 6 Flex system (Thermo Fisher Scientific, USA) using previously described methods (24). All swabs were run in duplicate, and the number of *Batrachochytrium* zoospore copies/μl was calculated using serial dilutions of synthetic *Bsal* and *Bd* DNA.

After necropsy, samples were preserved in 10% neutral buffered formalin until processing (2–364 days). The length of formalin storage was varied to examine potential effects of fixation time on ISH sensitivity. Animals were then decalcified in 0.5M EDTA, ph 8.0 for ~24 h. Serial transverse sections of the body and sagittal sections of all limbs and the tail were taken and submitted for routine processing and paraffin embedding.

### Naturally Occurring Little Devil Poison Frog (*Oophaga sylvatica*) Chytridiomycosis

A captive born and bred little devil poison frog (*Oophaga sylvatica*) was submitted to the Aquatic, Amphibian, and Reptile Pathology Service at the University of Florida's College of Veterinary Medicine for diagnostic necropsy. The frog arrived in 70% ethanol, was transferred to 10% neutral buffered formalin for 24 h, and then decalcified in 0.5M EDTA, ph 8.0 for ~24 h. Serial transverse sections of the body and sagittal sections of all limbs and the tail were taken and submitted for routine processing and paraffin embedding. Histopathologic findings were consistent with *Bd* chytridiomycosis, and the sample was utilized for ISH as an anuran (frog) chytridiomycosis sample.

### RNAScope^®^ Dual-Plex Chromogenic *in situ* Hybridization (ISH)

Custom RNAScope^®^ target-specific oligonucleotide (ZZ) probes were designed by Advanced Cell Diagnostics (Advanced Cell Diagnostics, Newark, California). Two ZZ probes, complementary to nucleotides 452-555 of *B. dendrobatidis* JEL 197 (NG_027619.1) and nucleotides 444-584 of *Batrachochytrium salamandrivorans* isolate AMFP (KC762293.1) 28S rRNA sequences, were designed each for *Bd* and *Bsal*, respectively. Chromogenic ISH was performed on a Leica BOND RX Fully Automated Research Stainer (Leica Biosystems, Buffalo Grove, Illinois) using RNAscope technology (22). Formalin-fixed paraffin-embedded tissue blocks were sectioned at 4 μm and mounted on Fisherbrand SuperFrost Plus glass slides (Fisher Scientific, Pittsburgh, PA). Automated, dual-plex, chromogenic RNAscope using methodology based on Anderson et al. (20) was performed using both *Bd* [LS, catalog #527278] and *Bsal* [LS, catalog #527288-C2] ISH probes. Pretreatment, hybridization, signal amplification, and detection (RNAscope Reference Guide, ACD/Biotechne) were performed on the Leica BOND RX. Pretreatment conditions included sequential deparaffinization, target retrieval using Leica Epitope Retrieval Buffer at 95°C for 15 m, protease digestion at 40°C for 15 min, and endogenous enzyme block. *Bd* and *Bsal* probes were hybridized at 42°C for 120 min, followed by signal development. After hybridization, serial signal amplification reactions were followed by online fast red chromogenic development of the *Bsal* probe. PermaGreen/HRP (Diagnostic Biosystems, Pleasanton, CA) was manually applied for 3 min to visualize the *Bd* probe. Slides were counterstained with hematoxylin, ImmunoHisto-Sealer (Diagnostic BioSystems, Pleasonton, CA) was applied for long-term retention of the green signal, and coverslips were mounted with Vectamount Permanent (Vector Laboratories, Burlingame, CA). The dihydrodipicolinate reductase (dapB) probe [LS, catalog #320758] was used as a negative control probe and was applied to separate histologic sections processed concurrently and in the same manner as those that received the *Bd* and *Bsal* probes. Glass slides were visualized on an Olympus BX43 microscope (Olympus Corporation, Tokyo, Japan); photomicrographs were captured with a Spot Insight 12 Mp sCMOS Color Camera (Spot Imaging, Sterling Heights, Michigan) and SPOT Imaging software (v5.4.3; Spot Imaging, Sterling Heights, Michigan).

## Results

### Specificity of *Bd*/*Bsal* ISH on Cultured *Batrachochytrium*

**Figure 1:**
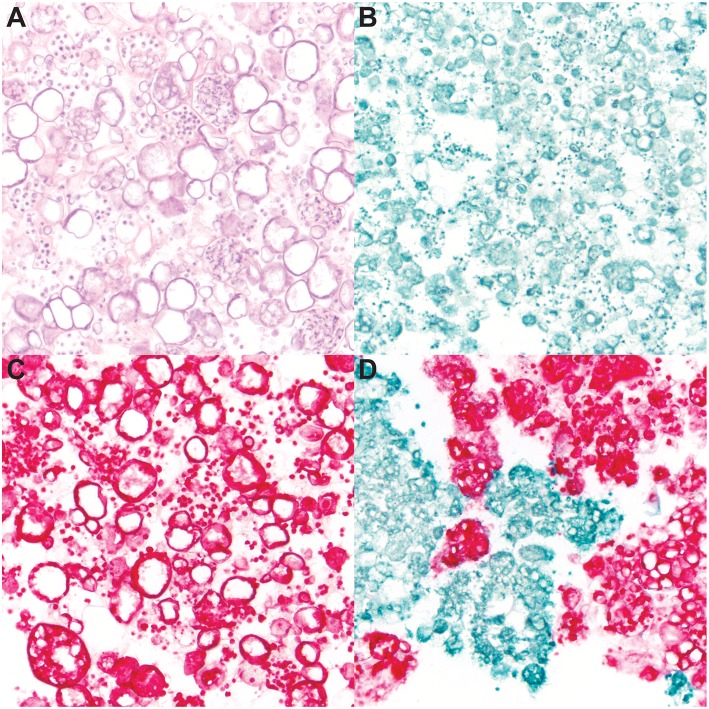
RNAScope^®^ *in situ* hybridization (ISH) of cultured *Batrachochytrium dendrobatidis* (*Bd*) and *B. salamandrivorans* (*Bsal*). Ethanol fixed samples of mixed *Bd*/*Bsal* **(A,D)**, *Bd* alone **(B)**, or *Bsal* alone **(C)** were suspended in Histogel sample processing matrix and processed for histology. Samples were then stained with hematoxylin and eosin **(A)** and dual-plex chromogenic *Bd/Bsal* RNAScope^®^ ISH targeting the 28S rRNA gene. *Bd* probe binding is visualized by green staining **(B,D)** and *Bsal* probe binding is visualized by red staining **(C,D)**. Intact fungal sporangia containing zoospores, empty sporangia, and free zoospores are present in all samples. All images are at 600x magnification.

To determine the specificity of the dual-plex chromogenic ISH assay, staining of *Bd* and *Bsal* cultures was performed (Figure 1), including hematoxylin and eosin (Figure 1A) which did not differentiate both fungi in mixed samples due to similar morphologic features. Pure cultures of *Bd* (Figure 1B) and *Bsal* (Figure 1C) demonstrated strong, positive green or red staining, respectively, of all fungal life stages including zoospores, mature zoosporangia, and empty, discharged sporangia. In a mixed sample, *Bd* and *Bsal* organisms were clearly differentiated by ISH (Figure 1D), with little to no cross reactivity or non-specific background staining. No positive staining was noted in the dapB stained negative control slides (data not shown).

### Specificity of *Bd/Bsal* ISH on Experimentally Infected Eastern Newts (*Notophthalmus viridescens*)

**Figure 2:**
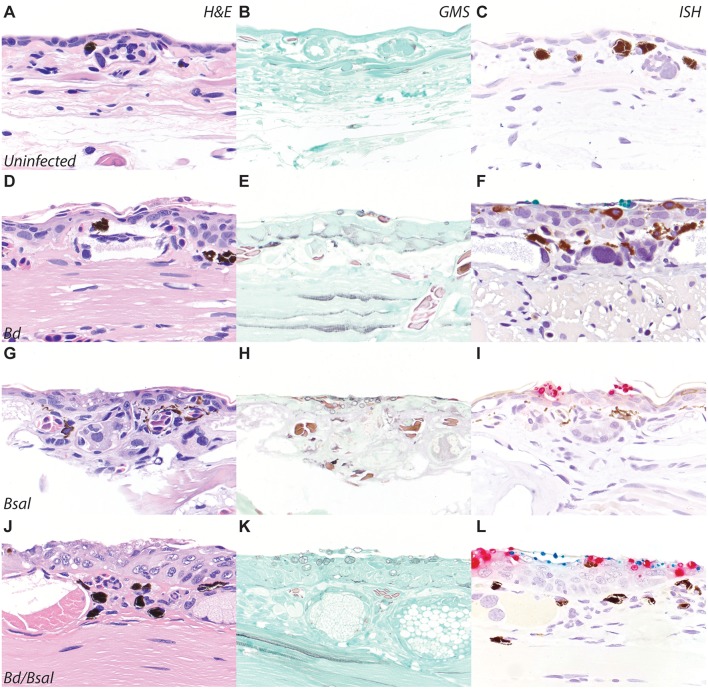
*Bd*/*BSal* RNAScope^®^ ISH of eastern newts (*Notophthalmus viridescens*). Skin sections from uninfected **(A–C)**, *Bd*-infected **(D–F)**, *Bsal*-infected **(G–I)**, or *Bd*/*Bsal* infected **(J–L)** are stained with hematoxylin and eosin **(A,D,G,J)**, Gomori methenamine silver to highlight fungal organisms **(B,E,H,K)**, or *Bd*/*Bsal* ISH **(C,F,I,L)**. *Bd* probe binding is visualized by green staining **(F,L)** and *Bsal* probe binding is visualized by red staining **(I,L)**. In all cases of chytridiomycosis, the presence of fungal organisms is accompanied by epidermal hyperplasia and hyperkeratosis. All images are at 200x magnification.

To confirm *Bd* and *Bsal* ISH probe reactivity on formalin-fixed paraffin-embedded tissue sections, automated dual-plex chromogenic ISH was carried out on slides of *N. viridescens* experimentally infected with *Bd* and/or *Bsal* (23). Newts were preserved with limited formalin fixation time (~48 h) before transfer to 70% ethanol for long term storage, and as such were ideal for RNAScope^®^ ISH validation per manufacturer's recommendations. Dual *Bd*/*Bsal* infected salamanders provided material to assess diagnostic specificity of the probe, and the small size of the newts also permitted screening of all tissues to assess for non-specific probe binding (Figure 2). Skin sections from uninfected newts (Figures 2A–C) stained with H&E, GMS, and ISH served as controls. *N. viridescens* infected with *Bd* alone, *Bsal* alone, or both *Bd*/*Bsal* all showed histopathologic changes consistent with chytridiomycosis, including epidermal hyperplasia, hyperkeratosis, and intralesional fungus (Figures 2D,G,J). *Batrachochytrium* zoosporangia and zoospores were absent in uninfected animals (Figure 2B), but present in all infected salamanders as highlighted by GMS staining (Figures 2E,H,K). No ISH reactivity was detected in the skin of *Bd*-/*Bsal*- newts (Figure 2C). In *Bd*+/*Bsal*- newts, zoosporangia exhibited green *Bd* probe staining (Figure 2F), while only red *Bsal* probe staining was detected in the fungus present within *Bd*-/*Bsal*+ newts (Figure 2I). In dual infected animals, both red *Bsal* and green *Bd* probe staining was present throughout the skin sections, and in some foci, *Bd*, and *Bsal* zoosporangia were intermingled amongst each other (Figure 2L). Mature fungal zoosporangia with a discharge tube, morphologically consistent with *B. dendrobatidis*, were highlighted by green *Bd* probe staining, while zoosporangia with prominent, multifocal septation (colonial zoosporangia), morphologically consistent with *B. salamandrivorans*, were highlighted by red *Bsal* probe staining (1, 5, 6). No non-specific staining of the *Bd* probe was noted in any of tissues of the examined newts; mild non-specific staining of the *Bsal* probe was noted in the retina and occasionally arthropod chitin (ingesta) in the gastrointestinal tract (data not shown). DapB control probe staining was uniformly negative in all animals (Supplementary Figure 1A).

### Validation of *Bd/Bsal* ISH in Other Amphibian Species

**Figure 3:**
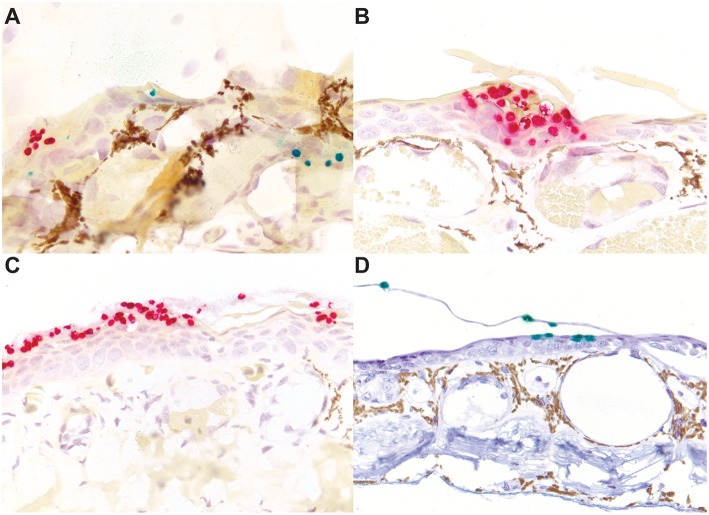
*Bd*/*BSal* RNAScope^®^ ISH of skin sections of mixed species with chytridiomycosis. Concurrent *Bd* and *Bsal* chytridiomycosis in an experimentally infected red-legged false brook salamander (*Aquiloeurycea cephalica*; **A**). *Bsal* chytridiomycosis in an experimentally infected large-blotched ensatina (*Ensatina eschscholtzii klauberi*; **B**) and a red salamander (*Pseudotriton ruber*; **C**). *Bd* chytridiomycosis in a naturally infected little devil poison frog (*Oophaga sylvatica;* **D**). *Bd* probe binding is visualized by green staining **(A,D)** and *Bsal* probe binding is visualized by red staining **(A–C)**. The golden tinctorial property of (**A–C**) is an artifact of the counterstain occasionally seen. **(A,B)** are at 400x magnification; **(C,D)** are at 200x magnification.

Four additional species of salamander and one frog species were used for automated dual-plex chromogenic *Bd/Bsal* ISH testing to confirm probe specificity in other amphibian species. Red-legged false brook salamanders (*A. cephalica*; *n* = 3), large-blotched ensatinas (*E. e. klauberi; n* = 5), yellow-eyed ensatinas (*E. e. xanthoptica*; *n* = 6), red salamanders (*P. ruber*; *n* = 3), and a little devil poison frog (*O. sylvatica*; *n* = 1). All salamanders (*n* = 17) were experimentally infected with *Bsal*, and necropsy fungal loads ranged from 1.4 × 10^2^ to 2.1 × 10^5^ genome copies per microliter (Table 1). The three *A. cephalica* were naturally infected with *Bd* at collection, with necropsy *Bd* fungal loads ranging from 6.2 × 10^2^ to 2.2 × 10^6^ (Table 1). No PCR was performed on the poison frog with chytridiomycosis on diagnostic necropsy. Positive ISH staining was observed in all species tested (Figure 3). Of the 17 qPCR *Bsal* positive salamanders, 16 salamanders were *Bsal* ISH positive (94.1%; Figures 3A–C). The *Bsal* ISH negative animal had the second lowest *Bsal* load of animals tested (1.4 × 10^2^ copies/μl). Of the 3 qPCR *Bd* positive salamanders, 2 salamanders were *Bd* ISH positive (66.7%; Figure 3A). The *Bd* ISH negative animal had the lowest *Bd* load of animals tested (6.2 × 10^2^ copies/μl). Both salamanders with discordant qPCR/ISH results were of the same species (*A. cephalica*) and had a formalin fixation time of 154 days (Table 1). However, positive *Bd* and *Bsal* probe staining was noted in one *A. cephalica* with higher fungal loads (Figure 3A). Both *Bsal* and *Bd* probe staining, when present, was strong, even in animals stored in formalin for up to 154 days. The one frog tested exhibited positive *Bd* probe staining (Figure 3D). Occasional non-specific staining of the retina and ingested chitin was noted as for *N. viridescens*. DapB control probe staining was uniformly negative in all animals (Supplementary Figures 1B–D and data not shown).

**Table 1:** Summary of animals used for *Bd*/*Bsal* RNAScope^®^ ISH validation.

**Species**	**Days in Formalin**	**qPCR loads (copies or zoospores/μL)**		**ISH**	
		***Bd***	***Bsal***	***Bd***	***Bsal***
Eastern newt (*Notophthalmus viridescens*)	2	–	–	–	–
	2	–	–	–	–
	2	70,851^Z^	–	+	–
	2	33,608^Z^	–	+	–
	2	–	268,366^Z^	–	+
	2	–	103,125^Z^		
	2	43,850^Z^	108^Z^	+	+
	2	140,944^Z^	59,838^Z^	+	+
	2	–	4,003^C^	–	+
	331	–	4,687^C^	–	+
	2	–	10,690^C^	–	+
	2	–	12,892^C^	–	+
	364	–	22,316^C^	–	+
	8	–	118,881^C^	–	+
	7	–	186,371^C^	–	+
	8	–	292,173^C^	–	+
Red-legged false brook salamander (*Aquiloeurycea cephalica*)	154	182,448^C^	208,652^C^	+	+
	154	2246,063^C^	139^C^	+	–
	154	622^C^	54,748^C^	–	+
Large-blotched ensatina (*Ensatina eschscholtzii klauberi*)	2	–	44,132^C^	–	+
	2	–	70,830^C^	–	+
	2	–	163,558^C^	–	+
	131	–	5,252^C^	–	+
	104	–	8,488^C^	–	+
Yellow-eyed ensatina (*Ensatina eschscholtzii xanthoptica*)	104	–	432^C^	–	+
	104	–	1,095^C^	–	+
	104	–	1,963^C^	–	+
	104	–	2,634^C^	–	+
	2	–	29,886^C^	–	+
	2	–	92,294^C^	–	+
Red salamander (*Pseudotriton ruber*)	120	–	70,723^C^	–	+
	120	–	87,258^C^	–	+
	120	–	234,771^C^	–	+
Little devil poison frog (*Oophaga sylvatica*)	1	*NP*	*NP*	+	–

*Length of tissue storage time in formalin prior to processing, quantitative PCR loads of Bd ± Bsal, and Bd/Bsal ISH results are shown*.

Z, zoospore equivalents;

C, *copies; NP, Not performed*.

### Effect of Formalin Fixation Time on *Bd/Bsal* ISH

Given the finding that animals with formalin fixation times up to 154 days could still exhibit positive *Bd/Bsal* ISH staining, two additional eastern newts (*N. viridescens*) with formalin fixation times of 331 (*Bsal* load−4.7 × 10^3^) and 364 (*Bsal* load 2.2 × 10^4^) days, respectively, were tested. Both animals were *Bsal* ISH positive, with no dramatic difference in staining distribution and intensity (data not shown).

### Sensitivity of *Bd/Bsal* ISH

To approximate the sensitivity of *Bd/Bsal* ISH in comparison to qPCR *Bd*/*Bsal* loads in salamanders, staining results from 33 salamanders representing 5 species were compared. *Bsal* loads ranged from 1.1 × 10^2^ to 2.7 × 10^5^ (zoospore equivalents) or 1.4 × 10^2^ to 2.9 × 10^5^ (ITS-1 copies) per microliter. Positive *Bsal* probe staining was present in tissue sections of animals with qPCR *Bsal* levels as low as 1.1 × 10^2^ zoospore equivalents (formalin fixation time, 2 days) or 4.3 × 10^2^ ITS-1 copies (formalin fixation time, 104 days) per microliter. *Bd* loads ranged from 3.4 × 10^4^ to 1.4 × 10^5^ (zoospore equivalents) or 6.2 × 10^2^ to 2.2 × 10^6^ (ITS-1 copies) per microliter. Positive *Bd* probe staining was present in tissue sections of animals with qPCR *Bd* levels as low as 3.4 × 10^4^ zoospore equivalents (formalin fixation time, 2 days) or 1.8 × 10^5^ ITS-1 copies (formalin fixation time, 104 days) per microliter.

### Distribution of *Bd/Bsal* Organisms in Tissue

**Figure 4:**
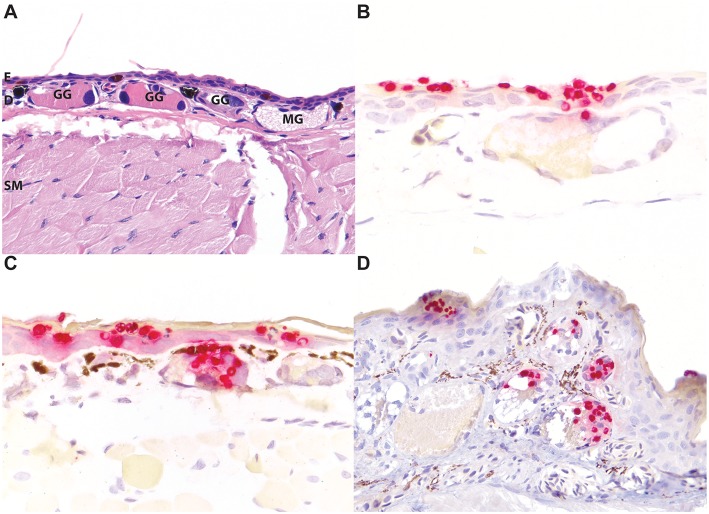
*Bsal* infection of dermal glandular epithelial cells in salamanders. Normal, uninfected eastern newt (*Notophthalmus viridescens*) skin stained with hematoxylin and eosin highlighting the epidermis [E], dermis [D], dermal glands (granular glands [GG] and mucus glands [MG]), and subcuticular skeletal muscle [SM] **(A)**. In experimentally infected red salamanders (*Pseudotriton ruber*; **B**), yellow-eyed ensatinas (*Ensatina eschscholtzii xanthoptica;* **C**), and large-blotched ensatinas (*E. e. klauberi*; **D**), positive red ISH staining of *Bsal* organisms extends down from the epidermis into the epithelial cells of dermal glands. In the most severely affected regions, abundant organisms may be present in multiple glands **(C)**. **(A,D)** are at 200x magnification; **(B,C)** are at 400x magnification.

The high contrast of particularly the red staining *Bsal* organisms, but also the green staining *Bd* organisms, in this assay afforded additional advantages for the identification of even low numbers of zoosporangia in tissues over routine and histochemical stains (Figure 2). The distribution of *Bd* and *Bsal* was compared in sections stained of all 32 positive amphibians in this study. *Bd* organisms were present primarily within the stratum corneum (Figures 2F,L, 3A), as has been previously reported (5, 6). *Bsal* organisms were predominantly found in clusters or plaques that extended down to the deeper levels of the epidermis (Figures 2I,L, 3A–C). The highest distribution of lesions were present on the distal limbs, tail, and head (data not shown), though foci of *Bsal* chytridiomycosis could be found in the epidermis throughout the body, consistent with previous reports (1). In some animals, *Bsal* zoosporangia were noted to extend into the neck and secretory epithelial cells of underlying dermal glands (Figure 4). The density of glandular *Bsal* zoosporangia ranged from low (Figure 4B) to moderate (Figures 4C,D) within individual glands, and sometimes to multifocal glands within a small area of skin (Figure 4D). *Bsal* zoosporangia were not noted in any other epithelial cells of the body other than the epidermis and dermal glands.

## Discussion

Accurate diagnosis of chytridiomycosis due to *B. salamandrivorans* (*Bsal*) in free-ranging salamanders, particularly given the high potential for natural co-infections with *B. dendrobatidis* (*Bd*), may not always be possible with routine histopathology and PCR alone. For this reason, we developed an automated, dual-plex chromogenic RNAScope^®^ *in situ* hybridization (ISH) assay for rapid, accurate, and simultaneous differentiation and co-localization of *Bd* and *Bsal* in tissue section.

The developed assay was highly specific for both *Bd* and *Bsal* in comparison to each other and amphibian tissues, with minimal cross-reactivity to host tissues from five species of salamander and one species of frog. Though any staining should always be interpreted with appropriate controls, the minimal non-specific reactivity in the examined salamanders and frogs suggests it is unlikely for other species to be dramatically different. The noted non-specific reactivity of the *Bsal* probe to the retina and to arthropod ingesta should not impair interpretation of cutaneous lesions typical of chytridiomycosis.

The assay detected *Bsal* in samples that remained in fixative for up to 364 days and *Bd* in tissues that remained in fixative up to 154 days. Though manufacturer's recommendations for optimal storage and handling of tissues are either 70% ethanol indefinitely or formalin fixation for <48 h, the ability of this assay to detect *Bsal* and *Bd* after extended (>48 h) formalin fixation will make it more useful for surveillance projects when samples may be archived for some time before submission. *Bsal* ISH staining was positive in sample fixed in formalin for 2 days with 1.1 × 10^2^ zoospore equivalents/μl (postmortem skin swab) and 104 days with 4.3 × 10^2^ ITS-1 copies/μl (postmortem skin swab), but negative in a sample fixed in formalin for 154 days with 1.4 × 10^2^ ITS-1 copies/μl (postmortem skin swab). This indicates that the *Bsal* ISH can be highly sensitive even after extended fixation times. Moreover, the high contrast between the light blue hematoxylin counterstain and the red *Bsal* probe staining may make diagnosis easier than more traditional histochemical special stains (such as GMS or periodic acid Schiff). As such, ISH may be advantageous over routine and histochemical stains for the identification of organisms in low-level or subclinical *Bsal* infections as identified by molecular diagnostics. Interpretation of the sensitivity of the *Bd* ISH is more difficult, as it was not a primary focus in this study and there is limited load distribution in the examined samples, particularly at lower levels. There was no detection of *Bd* probe signal in *A. cephalica* with 6.2 × 10^2^ ITS-1 copies/microliter of a postmortem skin swab, suggesting a lower sensitivity of the dual assay for *Bd* than *Bsal*. However, more samples representing a wider range of fixation times and *Bd* loads are necessary for a more definitive *Bd* sensitivity.

Little modification of previously published techniques for automated dual-plex chromogenic RNAScope^®^ ISH was required (20, 22). Three chromogenic options are available for dual-plex RNAScope from the manufacturer: brown and green in channel one (*Bd* probe), red in channel two (*Bsal* probe). Due to the presence of abundant melanocytes in the amphibian epidermis, the HRP-based green chromogen as provided in the RNAScope^®^ LS Green Accessory pack was selected for visualization of *Bd* probe binding. However, rapid and significant fading and stain dissolution was noted starting as soon as 48 h after processing with this chromogen. For long-term retention of the green signal of the *Bd* probe, an alternative green chromogen (PermaGreen/HRP) and the use of an additional preservation solution (Immunohisto-Sealer) were used. Neither of these modifications had an effect on the *Bsal* probe fast red signal. The cost of performing this assay is not without consideration, at approximately $100–200 USD per slide, depending on single- or dual-plex and automated or manual staining. The use of ISH may not be appropriate or necessary in all instances. However, for high profile diagnostic cases it can provide important etiologic confirmation of *Batrachochytrium* infections, and for pathogenesis studies, it can provide specific pathogen/lesion co-localization.

One of the most noteworthy findings in this study was the identification of *Bsal* zoosporangia not only within the epithelial cells of the epidermis but within the epithelial cells of dermal glands. In *Bd* infections, zoosporangia invade mature cells of the epidermal stratum granulosum and stratum corneum, sparing the basal epidermis and any underlying structures (25, 26). Fungal infection can result in epidermal hyperplasia and hyperkeratosis significant enough to disrupt cutaneous electrolyte transport, gas exchange, and osmoregulation and cause death (27). The pathogenesis of *Bsal* chytridiomycosis, however, has not been elucidated. As the distribution of lesions associated with *Bsal* infection can be considerably patchier than the more generalized skin response in *Bd* infections, it is possible if not likely that *Bsal* mortalities involve a different underlying pathogenesis. There are two primary types of dermal glands in amphibians: mucus and granular (serous or poison) glands (Figure 4A). The glands are divided into three parts: (i) the duct which is lined by modified keratinocytes and serves as the conduit from the gland to the epidermal surface; (ii) the intercalary region lined by reserve blastic epidermal cells; and (iii) the gland alveolus lined by secretory epithelium (28). While it would not be unexpected to see *Batrachochytrium* zoosporangia in the keratinized ducts of dermal glands (Figure 4B), in some animals fungal organisms extended down into the gland alveoli within secretory epithelial cells (Figures 4C,D). The presence of *Bsal* zoosporangia within glandular epithelial cells may affect the secretory function of these glands. Cutaneous glandular secretions assist in maintaining the skin as a moist surface for gas exchange, preventing water loss, and as a source of active and passive defense in the form of noxious or poisonous substances, or antimicrobial compounds (29–32). Additional studies to investigate the effects of *Bsal* infection on cutaneous mucus and granular gland function are needed to identify the role of the infection of these glands on the pathogenesis of *Bsal* infections.

This report describes the development and characterization of an automated, dual-plex, chromogenic RNAScope^®^ ISH assay for simultaneous detection and differentiation of *Bd* and *Bsal* in tissue sections. In combination with molecular diagnostics and routine histopathology, this assay represents an important tool for definitive diagnosis of *Bsal* chytridiomycosis. Such multimodal confirmation may be useful in high profile or complicated in diagnostic cases and/or surveillance studies. The assay also will also be a useful tool in the study of laboratory infections of *Bsal* in salamanders and other amphibian hosts to better understand the potential host range, tissue tropism, and pathogenesis of the emerging fungal disease of amphibians, as well as the potential interaction of *Bsal* and *Bd* in mixed infections.

## Data Availability

All datasets generated for this study are included in the manuscript/Supplementary Files.

## Ethics Statement

The animal study was reviewed and approved by University of Maryland IACUC protocol R-15-15. University of Tennessee Institutional Animal Care and Use Committee protocol #2395. Written informed consent was obtained from the owners for the participation of their animals in this study.

## Author Contributions

RO and SF designed the experiments. MB performed and optimized the ISH staining. RO and AT interepreted the ISH staining. AL, KL, DM, EC, and MG performed experimental infections of the animals. RO drafted the manuscript and created the figures. All authors reviewed the manuscript.

### Conflict of Interest Statement

The authors declare that the research was conducted in the absence of any commercial or financial relationships that could be construed as a potential conflict of interest.

## Supplementary Material

Supplementary Figure 1 DapB negative control staining of amphibian tissues. No positive signal is seen in DapB control staining of skin from an eastern newt (*Notophthalmus viridescens*; **A**), a yellow-eyed ensatina (*Ensatina eschscholtzii xanthoptica;* **B**), a red salamander (*Pseudotriton ruber*; **C**), or a little devil poison frog (*Oophaga sylvatica;* **D**). All images are at 400x magnification.
