# Supplementary figures and images for: Differentiating Batrachochytrium dendrobatidis and B. salamandrivorans in Amphibian Chytridiomycosis Using RNAScope® in situ Hybridization
Source: Front Vet Sci. 2019 Sep 12;6:304. doi: 10.3389/fvets.2019.00304 (PMC6751264; doi:10.3389/fvets.2019.00304)

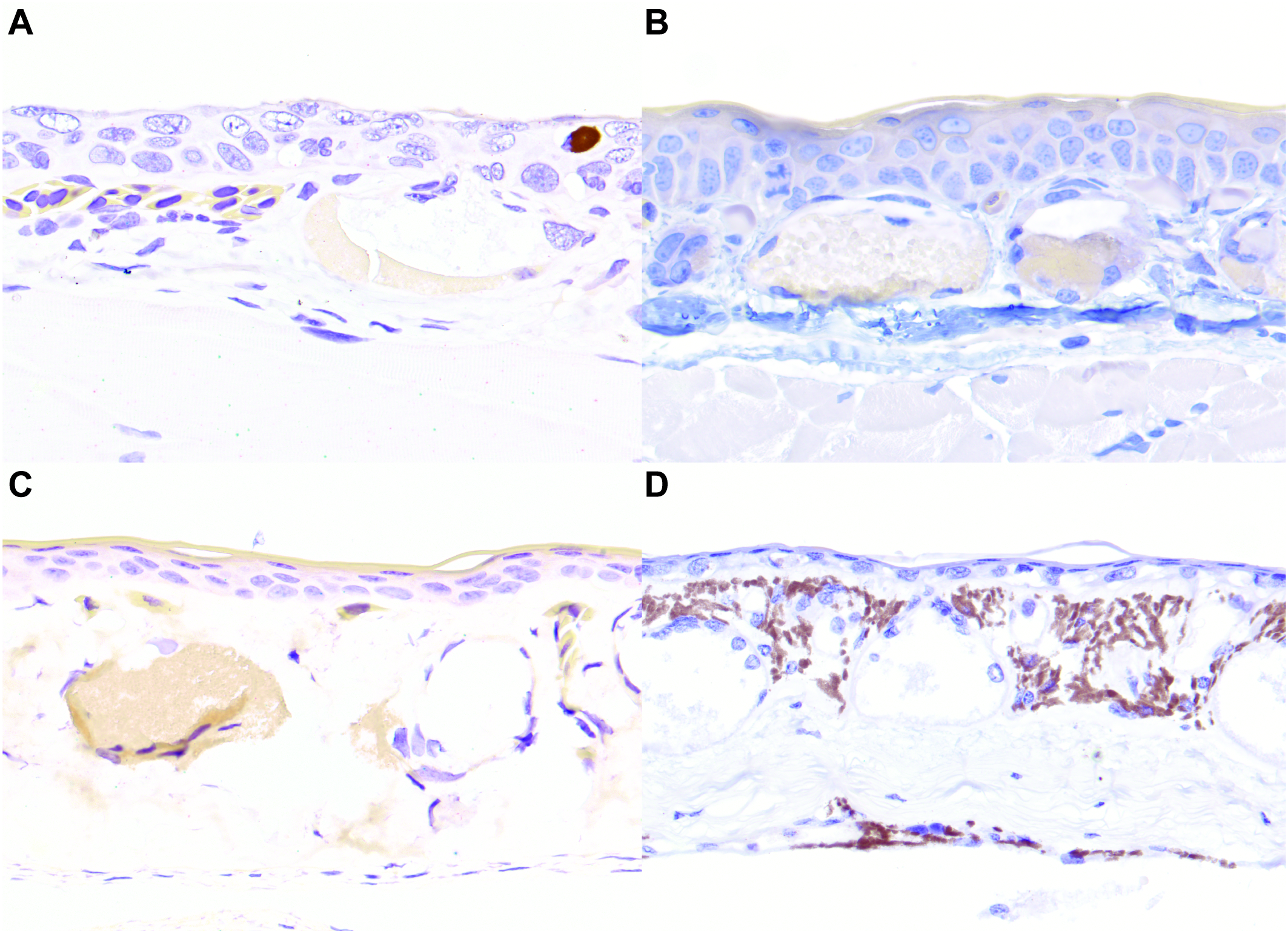

Supplement: Supplementary Figure 1 — DapB negative control staining of amphibian tissues. No positive signal is seen in DapB control staining of skin from an eastern newt (Notophthalmus viridescens; A), a yellow-eyed ensatina (Ensatina eschscholtzii xanthoptica; B), a red salamander (Pseudotriton ruber; C), or a little devil poison frog (Oophaga sylvatica; D). All images are at 400x magnification. [file Image_1.TIF]
